# The Structural Similarity Can Identify the Presence of Noise in Video Data from Unmanned Vehicles

**DOI:** 10.3390/jimaging11110375

**Published:** 2025-10-26

**Authors:** Anzor Orazaev, Pavel Lyakhov, Valery Andreev, Denis Butusov

**Affiliations:** 1 Department of Mathematical Modeling, North-Caucasus Federal University, Stavropol 355017, Russia; 2 North-Caucasus Center for Mathematical Research, North-Caucasus Federal University, Stavropol 355017, Russia; ljahov@mail.ru; 3 Computer-Aided Design Department, St. Petersburg Electrotechnical University “LETI”, 5 Professora Popova St., Saint Petersburg 197022, Russia; vsandreev@etu.ru

**Keywords:** image processing, image quality assessment, noise detection, structural similarity, unmanned vehicles, video processing

## Abstract

This paper proposes a method for detecting distorted frames in video footage recorded by an unmanned vehicle. The proposed detection method is performed by analyzing a sequence of video frames, utilizing the contrast aspect of the structural similarity index between previous and current frames. This approach allows for the detection of distortions in the video caused by various types of noise. The scientific novelty lies in the targeted adaptation of the SSIM component to the task of real interframe analysis in conditions of shooting from an unmanned vehicle, in the absence of a reference. The three videos were considered during the simulation. They were distorted by random significant impulse noise, Gaussian noise, and mixed noise. Every 100th frame of the experimental video was subjected to distortion with increasing density. An additional measure was introduced to provide a more accurate assessment of distortion detection quality. This measure is based on the average absolute difference in similarity between video frames. The developed approach allows for effective identification of distortions and is of significant importance for monitoring systems and video data analysis, particularly in footage obtained from unmanned vehicles, where video quality is critical for subsequent processing and analysis.

## 1. Introduction

Unmanned transport includes various types of autonomous vehicles such as unmanned cars, unmanned aerial vehicles (UAVs), autonomous ships, and trains, which are capable of operating without human involvement [1]. The use of video data obtained from unmanned vehicles (UVs) significantly enhances the capabilities of monitoring and analysis systems across various fields. UVs are equipped with high-quality cameras and sensors that can continuously and in real time capture video streams, ensuring accuracy and timeliness of information collection [2]. Each type of transport serves its specific purpose: unmanned cars are used for passenger and cargo transportation in urban environments, drones for goods delivery, aerial photography, and monitoring, autonomous ships for cargo transport and ocean research, and trains for efficient passenger and freight transport on rails [3]. All of these systems are aimed at improving safety, reducing costs, and enhancing logistics in various sectors [4].

Distortions occurring during the registration of video and photo data by ground-based unmanned vehicles reduce the quality of images, which play a key role in object recognition and environmental analysis. This can lead to errors in assessing the traffic situation, misidentification of pedestrians, vehicles, and traffic signs, thus increasing the risk of accidents [5]. This issue becomes especially critical under low-lighting conditions or in adverse weather. Additionally, digital noise can hinder the performance of machine vision algorithms and neural networks, slowing down the system’s response and reducing the accuracy of decision-making [6].

Digital noise in video recorded by ground-based unmanned vehicles represents visual distortions that occur due to insufficient lighting, high speeds, especially during sharp turns or changes in lighting conditions, and technical limitations of the camera, as well as interference from electromagnetic fields, which can further impact the recording quality [7]. This noise manifests as graininess or color artifacts. Filtering and post-processing algorithms are used to minimize digital noise, along with high-resolution cameras and enhanced light sensitivity. In real-time conditions, high-quality video with minimal noise is critically important for the proper functioning of navigation systems and ensuring the safety of unmanned vehicles.

The real-time denoising is a resource-intensive task. Additionally, there is no need to correct frames that are not distorted. Detecting frames affected by noise can help address these issues. This paper proposes a method in which distorted frames can be identified using the Structural Similarity Index Measure (SSIM). Similarity is determined between consecutive frames. It is assumed that with a sufficiently high frame rate, the difference between frames will be minimal. A divergence between two consecutive frames indicates the presence of distortions in the image, the operation of built-in digital filters, or high dynamics in the video. To exclude high dynamics in the video or other factors that do not affect image quality but still show high divergence between consecutive frames, the SSIM aspects of brightness, contrast, and structure were considered. The contrast aspect demonstrated the highest similarity between frames under natural conditions, without the presence of noise in the video. The decision on whether a frame is distorted is made by comparing the measure to a threshold value. The proposed method is deliberately designed as a simple yet effective solution for embedded drone systems where computational resources are strictly limited. Unlike complex neural network approaches, our method provides comparable accuracy with lower computational power requirements.

Our contribution is as follows:

1. A method based on a similarity measure for assessing the resemblance of video frames for detecting distorted frames is proposed.
2. A comparison of the proposed method with known image quality assessment methods has been conducted.
3. An additional sensitivity measure for detecting distorted frames has been developed.

It has been demonstrated that the method based on SSIM can identify the presence of noise in video data from unmanned vehicles.

The scientific novelty of the proposed method consists of adapting the classic SSIM for the task of detecting distorted frames in the video from unmanned vehicles, considering the contrast aspect, which is the most sensitive component. Unlike existing methods, the proposed approach does not require a reference image, has low computational costs and allows you to perform real-time detection. This makes it especially valuable for integrating it into the energy-limited systems, i.e., into ground or air UAVs, where the use of resource-intensive algorithms is unacceptable. The method also offers a simple threshold scheme of personnel classification.

The paper is organized as follows: Section 2 provides an overview of existing image similarity comparison methods. Section 3 discusses the proposed method for detecting distorted video frames. Then, Section 4 presents a comparison of the proposed distorted image detection method with the other similarity assessment methods. Section 5 discusses the obtained results. Section 6 concludes the paper.

## 2. Related Research

To our knowledge, the problem of detecting distorted frames in video (without a reference frame and in real time) in the presented form is considered for the first time, and related works considered in this section are not directly intended to solve this problem but solve similar problems of comparing frames in video. The task of detecting fakes and any other type of video manipulation based on frame comparison is similar to the task of detecting distorted frames, since in both cases it is necessary to identify anomalies that may indicate tampering with the original image or video [8]. A common approach in the detection of fake content is to analyze a sequence of frames to identify inconsistencies. These inconsistencies include unusual scene transitions, discrepancies in lighting, object movement, or the presence of editing artifacts. In both cases, the task boils down to detecting inconsistencies between video frames. This requires developing algorithms that are capable of identifying subtle changes in the video stream.

Feature-based methods for detecting distortions in video are an important part of research in digital forensics and image processing. These methods are actively used to extract, analyze, and compare specific features such as contours, textures, and angles between the original and distorted frames [9].

### 2.1. Methods for Detecting Fakes and Manipulated Video

In the field of fake and manipulation detection in video, feature-based methods are widely used. The SIFT method is one of the most popular algorithms for extracting and describing key points in images, which is applied to various computer vision tasks, including the detection of fakes and manipulation in video [10]. If an object was inserted or moved during editing, its key points will not match the real moving objects in other frames. In the work [11], the authors assess sensor noise based on a locally adaptive discrete cosine transform, then correlate the noise residue of the frame under investigation with the sensor noise and the noise residue of the previous frame to detect digital manipulation. The SURF method is an algorithm for extracting and describing features (key points) in images, developed as an improved version of the SIFT method [12]. The main goal of SURF is to increase processing speed while maintaining resistance to scale, rotation, and lighting changes. SURF is significantly faster than SIFT due to the use of Haar filters and a more efficient key point search [13]. Despite the improved performance of the real-time video processing, SURF requires significant computational resources, especially in cases of high resolution or long videos. The LBP method is an algorithm for texture analysis of images. LBP is based on transforming the image, making it suitable for analyzing local texture features [14]. The main idea of LBP is to encode the local texture by comparing each pixel with its neighbors. LBP histograms can be used for image classification or video change analysis. LBP is a fast and simple method that can be efficiently implemented for processing large volumes of data, making it suitable for real-time video analysis. However, LBP can be sensitive to noise in images, which may lead to reduced accuracy under poor video quality or images with a high level of noise [15].

The ORB method [16] is an algorithm for extracting and describing image features, combining a fast algorithm for key point detection [17] with an efficient method for key point description [18]. ORB was developed as a fast and efficient solution for tasks similar to those addressed by algorithms such as SIFT and SURF. It offers improved computational efficiency and is not subject to patents, making it convenient for use in commercial applications [19]. ORB is significantly faster than methods like SIFT and SURF due to the use of binary descriptors [20]. However, binary descriptors can be sensitive to noise, especially under poor video or image quality conditions.

### 2.2. Deep Learning and Neural Network Approaches

U-Net is commonly used for segmentation tasks, but it can also be applied to detect changes between two images, such as identifying pixel differences [21]. Three-dimensional CNN is an extension of standard convolutional networks, where a temporal axis is added. This allows the network to consider information from a sequence of frames, extracting both spatial and temporal features [22]. Three-dimensional convolutional layers apply filters that pass through the temporal axis of the video, thus considering changes between frames. These networks are capable of capturing both the structure of the objects and their dynamics over time. Recurrent neural networks, especially LSTM, are well-suited for tasks where it is important to account for long-term dependencies between video frames [23]. LSTM can analyze changes in the video by remembering information about previous frames and using this for prediction or change detection [24]. LSTM networks receive a sequence of frames and can detect changes as they occur, retaining information about previous frames in their memory [25].

FineVQ [26] has been proposed as a deep learning-based model designed to provide fine-grained video quality assessment across multiple dimensions, including noise, blur, artifact, and temporal quality. Unlike traditional approaches, FineVQ leverages a large-scale annotated dataset (FineVD) and employs instruction tuning and LoRA adaptation to deliver a unified model capable of scoring, rating, and describing video quality. This method demonstrates state-of-the-art performance on several UGC-VQA benchmarks due to its integration of spatial, motion, and linguistic features via multimodal learning. However, despite its high accuracy, FineVQ has limitations in practical deployment scenarios such as onboard processing in UAVs. The method requires significant computational resources, involves large pre-trained models, and is not designed for real-time inference or systems with limited hardware capabilities, which are critical constraints in UAV applications. In the context of adapting deep learning for specialized applications, transfer learning methodologies show significant potential, as demonstrated in work by [27], where pre-trained VGG-19 and ResNet-152 models were successfully applied to facial emotion recognition in healthcare systems. Despite varying performance across datasets, this approach confirms the importance of dataset-specific preprocessing and model optimization for particular domains, which aligns with our objective of developing a specialized solution for unmanned vehicle video analysis. The challenge of developing highly efficient yet accurate deep learning solutions for specialized visual analysis tasks is notably demonstrated in the structural health monitoring domain. Ref. [28] introduced SHSnet, an efficient, attention-based encoder–decoder network designed for the end-to-end semantic segmentation of complex, fine crack patterns in Engineered Cementitious Composites. Their model achieves high performance (e.g., 0.84 F1-score) while requiring an order of magnitude fewer computational parameters than other models in the literature. A significant practical outcome is that the automated analysis with SHSnet achieved equivalent accuracy to manual microscopy but required approximately 100 times less processing time [29]. This successful development underscores a critical balance between computational efficiency and analytical precision, a design philosophy that directly aligns with the objectives of our work for real-time video distortion detection in resource-constrained unmanned vehicle systems. Addressing feature extraction challenges in suboptimal imagery remains critical across computer vision domains. In infrared target detection, where high noise levels and poor texture information limit conventional CNNs, ref. [30] developed a dual-domain network combining frequency and spatial processing with resource-adaptive feature allocation. Their method achieved substantial performance improvements (8.05–10.14% mAP) by designing specialized architecture components for handling domain-specific limitations. This demonstrates the effectiveness of targeted feature enhancement strategies, which resonates with our approach of optimizing the contrast component of SSIM for efficient distortion detection in UAV video streams under computational constraints. The pursuit of efficient model architectures that maintain high performance under resource constraints represents a significant trend in practical computer vision applications. The FasterSal network for RGB-D salient object detection [31] exemplifies this direction by replacing conventional dual-stream designs with an efficient single-stream structure that processes both RGB and depth modalities. This architectural optimization achieves an impressive balance of 63 FPS on CPU with only 3.4 million parameters while maintaining competitive accuracy, demonstrating that careful feature integration strategies can overcome the computational burdens of multi-modal processing. This approach resonates with our methodology of selectively leveraging the most informative component of structural similarity for distortion detection, rather than employing computationally expensive full metric calculations in UAV video analysis systems.

### 2.3. Image and Video Quality Assessment Methods

There are various methods for assessing image quality that allow for the evaluation of consecutive video frames [32]. These measures are divided into NR-metrics, which do not require a reference image, and FR metrics, where the reference image is the previous video frame [33]. NR-metrics are useful in real-world applications where a reference is unavailable or difficult to compute. One such measure is NIQE, based on natural image statistics [34]. NIQE uses a model of the statistical properties of natural images, such as texture, color, and structure [35]. NIQE can assess how much an image deviates from the characteristics of natural images, providing a quality score [36]. The BRISQUE measures image quality by using statistical characteristics of local and global structures within the image [37]. BRISQUE evaluates the degree of image distortion by comparing it to typical characteristics of natural images [38]. PIQE is another NR-metric focused on perception. It takes into account both typical distortions (such as noise or loss of sharpness) and more subtle distortions that may be noticeable to the human eye. PIQE aims to align with human visual perception, providing a quality assessment based on real human preferences [39]. The Light Field Image Quality Assessment (LFIQA) method [40] is based on the use of 3D shearlet transform and tensor color domains. LFIQA is a comprehensive approach to assessing the quality of light field images, which combines high processing speed and assessment accuracy. The BHSE-VQA method [41] is based on modeling bidirectional perception in the human visual system, accounting for both bottom-up (feedforward) and top-down (feedback) processes. It employs multi-level spatio-temporal feature extraction, redistribution of feature weights to reflect hierarchical perception, and temporal attention for accurate quality assessment of user-generated videos. NR-metrics are not capable of correctly identifying distorted frames. It is assumed that a frame distorted by noise will receive low measure scores [42].

FR metrics use pairwise comparison of an image with the original, which allows for assessing how much the image is distorted relative to the reference [43]. PSNR is one of the simplest and most frequently used image quality methods. It measures the ratio of the signal power (the original) to the noise power (distortions) in an image [44]. However, this measure does not always correlate with human perception. Images with high PSNR values can appear poor [45].

An important condition for using FR metrics to detect distorted video frames is the absence of frame stitching and a sufficiently high frame rate, where each subsequent frame will contain partial information from the previous frame [46]. The SSIM measures structural similarity between images, evaluating changes in structure, texture, and contrast [47]. This method attempts to more accurately model human image perception than PSNR. The work in [48] proposes a method for analyzing video data recorded by an unmanned aerial vehicle, using the Structural Similarity Index for evaluation. This method successfully detects delays, frame distortions, and dynamic changes in the video scene. The VMAF is widely used for assessing video quality in the compression and streaming scenarios, as well as for comparing different video codecs and compression settings [49]. VMAF uses several methods to assess video quality, which are combined into a single metric. These methods include both traditional approaches and machine learning techniques. Like many machine learning methods, VMAF requires high-quality training data and may not always perform accurately in conditions that differ significantly from the training samples [50]. Additionally, VMAF requires substantial computational resources, making it less suitable for real-time use, especially for mobile devices and applications with limited computational power [51]. Surveys in perceptual video quality assessment, such as the comprehensive work [52], emphasize the evolution from traditional knowledge-driven models (e.g., SSIM) to advanced data-driven approaches using deep learning. These models show strong performance on complex, semantically rich content and application-specific domains such as VR and streaming. However, the high computational cost and dependency on large-scale training datasets limit their applicability in real-time, resource-constrained environments such as UAVs. In contrast, our proposed method leverages the robustness of the SSIM framework while simplifying it by isolating and enhancing the contrast component, which is highly sensitive to noise and distortion in video frames. This design choice enables fast, accurate, and reference-free detection of visual anomalies in real-world UAV footage, making the method especially valuable for onboard quality control in embedded systems.

### 2.4. Scene Change Detection Methods

The task of detecting corrupted frames in video is largely similar to the task of scene change detection, as both require comparing frames based on visual similarity metrics such as SSIM. In both cases, the key step is to analyze changes between consecutive frames: abrupt or gradual deviations may indicate either a scene change or distortions (e.g., compression artifacts, noise, or technical defects).

In [53], a method for scene change detection in video based on SSIM is presented. The authors note that existing algorithms struggle with gradual transitions between scenes and propose a new approach for analyzing the dynamics of SSIM value sequences. To improve detection accuracy, a statistical measure of sequence variability is introduced. Experiments confirm the method’s robustness to noise and interference.

In [54], a computer vision-based automated inventory management system for supermarkets is proposed. One of the key components of the system is an SSIM-based scene change detection algorithm, which is used to detect human presence. Additionally, object detection methods are applied to count items on shelves. If the number of items falls below a specified threshold, the system sends a notification to the responsible person. For convenient product identification, optical character recognition (OCR) is used.

The next section will discuss the proposed method based on the contrast aspect of the SSIM. An important factor is the ability to use the proposed method in real-time. The proposed method allows for the accurate detection of distorted frames with minimal computational cost.

## 3. Proposed Method for Detecting Distorted Video Frames

High-quality sensors are used for video recording on unmanned vehicles, ensuring filming in dynamic scenes. Video recording on unmanned vehicles considers factors such as changes in lighting, the movement of the device itself, and surrounding objects. Cameras typically have automatic systems for adjusting exposure, focus, and contrast, which help maintain high image quality for unmanned vehicles [55].

Let two consecutive frames of the video be obtained, where *i* is the current frame of the video, and (*i* − 1) is the previous frame of the video. The *SSIM*, as a similarity measure between video frames, indicates changes in brightness, contrast, and structure that occurred during the time interval between the frames and is calculated using the following formula:

(1) $$SSIM(i-1,i)={\left[lum(i-1,i)\right]}^{\alpha }\cdot {\left[contrast(i-1,i)\right]}^{\beta }\cdot {\left[struct(i-1,i)\right]}^{\gamma },$$

where

(2) $$lum(i-1,i)=\frac{2\mu_{i-1}\mu_{i}+C_{1}}{\mu_{i-1}^{2}+\mu_{i}^{2}+C_{1}},$$

(3) $$contrast(i-1,i)=\frac{2\sigma_{i-1}\sigma_{i}+C_{2}}{\sigma_{i-1}^{2}+\sigma_{i}^{2}+C_{2}},$$

(4) $$struct(i-1,i)=\frac{\sigma_{i(i-1)}+C_{3}}{\sigma_{i-1}\sigma_{i}+C_{3}},$$

where $\mu_{i-1},\mu_{i}$ is the local mean values; $\sigma_{i-1},\sigma_{i}$ are the standard deviations; $\sigma_{i(i-1)}$ is the cross-covariance for frames *i* − 1 and *i*; lum,contrast,struct refers to the similarity in brightness, contrast, and structure, respectively; α,β,γ are the coefficients of lum,contrast,struct influence, respectively, $C_{1},C_{2},C_{3}$ are constants.

Typically, a simplified SSIM formula is used, where the condition α=β=γ=1 and $C_{3}=C_{2}/2$ holds. The simplified SSIM formula is as follows.

(5) $$SSIM(i-1,i)=\frac{\left(2\mu_{i-1}\mu_{i}+C_{1})(2\sigma_{i(i-1)}+C_{2}\right)}{\left(\mu_{i-1}^{2}+\mu_{i}^{2}+C_{1})(\sigma_{i}^{2}+\sigma_{i-1}^{2}+C_{2}\right)}.$$

When analyzing the changes in the video under certain conditions, the optimal detection strategy is to isolate the factor that induces the most severe deviation in the SSIM metric. On the other hand, the difference between the frames can be described as

(6) SSIM(i−1,i)=D⋅N⋅F⋅O.

where *D* is the influence of the difference between frames caused by object movement, the movement of the recording device, changes in lighting, and other natural factors affecting pixel mismatches (this characteristic depends on the speed of the unmanned vehicle as well as the frame rate of the video); *N* is the influence of noise occurring in the images; *F* is the influence of digital noise reduction filters or other embedded algorithms; *O* is the influence of various control elements or information overlaid on the recorded video frames, such as the OSD (On-Screen Display) technology. The OSD can be static, such as a display of current settings, or dynamic, such as prompts that appear depending on user actions.

**Figure 1:**
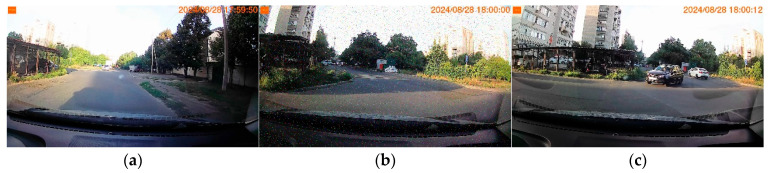
Frames of the experimental video (Example 1): (**a**) Frame 1. (**b**) Frame 300 (noise impact). (**c**) Frame 650.

**Figure 2:**
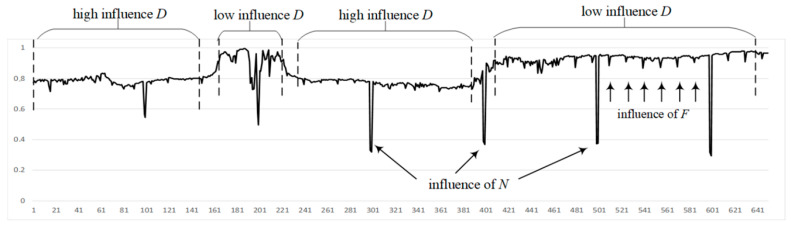
Influence of different factors on frame similarity measured by SSIM: D—object movement, N—noise, F—digital filters, O—OSD elements.

**Example 1.**  *Consider an experimental video recorded on the model of the car recorder MiDriveD01, which contains 650 frames; frame resolution is 1920 × 1080; frame rate is 30; video format is mp4; the video frames contain OSD information. The first, last, and an example frame affected by N are shown in Figure 1. Figure 2 presents a video fragment indicating the influences of D, N, F, and O using SSIM. The video is subject to periodic influence from F, which is caused by the recorder’s algorithm features. Every hundred frames, the influence of N is visibly noticeable, and these distortions are artificially introduced. The figure also shows a clear example of the strong influence of D; the influence of O on this video is not significant, which is due to the high resolution of the video frames.*

At a sufficiently high frame rate, the difference between frames *SSIM*(*i* − 1, *i*) will be low, and the SSIM values will be high. To determine the influence of *D*, *N*, *F*, and *O* on *SSIM*(*i* − 1, *i*), it is necessary to introduce a threshold value *T*. Using *T*, it can be concluded whether *N* has caused low similarity between the images.

**Figure 3:**
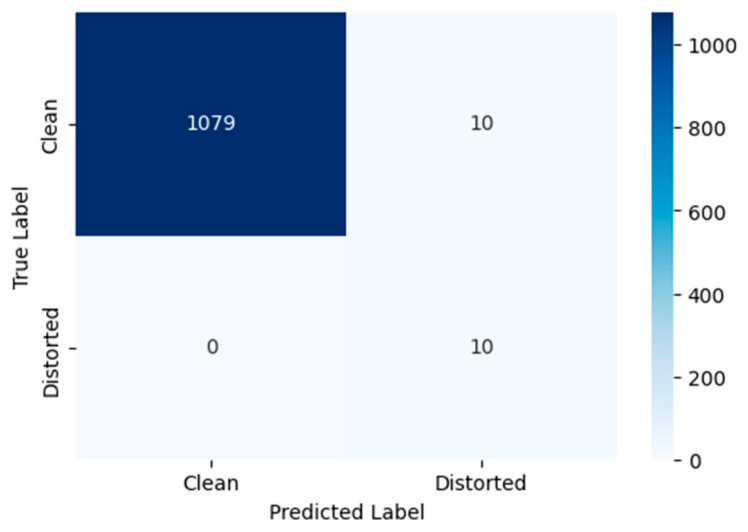
Confusion matrix for the binary classification of video frames as “distorted” or “clean” using the proposed method with a threshold of *T* = 0.9.

Standard classification metrics, such as precision, recall, F1-score, and overall accuracy, were used to quantitatively evaluate the efficacy of the chosen threshold *T* for detecting distorted frames. The calculation of these metrics is based on the analysis of the confusion matrix, which takes into account True Positive (TP), False Positive (FP), True Negative (TN), and False Negative (FN) detections. Figure 3 presents the confusion matrix for the binary classification of video frames as “distorted” or “clean”, using the proposed method with a threshold of *T* = 0.9. The values of the metrics Accuracy, Precision, Recall, F1-Score are presented in Table 1.

The choice of the threshold *T* = 0.9 was aimed to ensure maximum detection recall (Recall = 1.0), which guarantees the identification of all distorted frames—a critical requirement for subsequent analysis in unmanned vehicle systems. This configuration achieved an overall classification accuracy of 99.1%. The Precision value of 0.5 indicates that frames immediately following distorted ones differ significantly and are consequently classified as distorted by the proposed method. This observation is supported by the confusion matrix, where FP = TP. The F1-score of 0.667 confirms the method’s operational reliability under the chosen threshold, which aligns with a conservative detection strategy. The performance metrics are identical for Video 1, Video 2, and Video 3.

**Figure 4:**
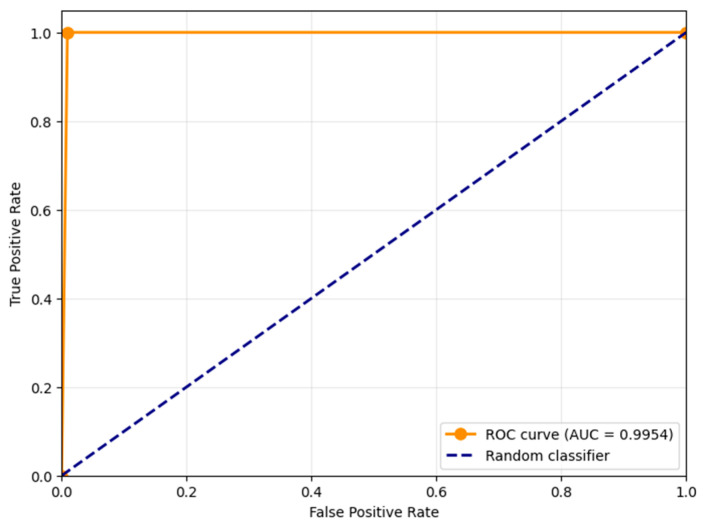
Receiver Operating Characteristic (ROC) curve for the proposed distortion detection method, with the Area Under the Curve (AUC).

Figure 4 shows the Receiver Operating Characteristic (ROC) curve for the proposed distortion detection method, with the Area Under the Curve (AUC) value of 0.9954. This near-perfect AUC score demonstrates the exceptional capability of the proposed contrast-based measure *S* to discriminate between distorted and clean frames across all possible classification thresholds. The ROC curve’s strong performance, hugging the top-left corner of the plot, indicates that the method maintains high true positive rates while keeping false positive rates low throughout the operating range. This outstanding separation capability confirms that the contrast component of SSIM serves as a highly effective feature for detecting noise-induced distortions in UAV video sequences, providing robust performance regardless of the specific threshold choice.

When determining frame distortion in the video, the most important factor is the influence of *N*. Therefore, it is necessary to select a measure where the influence of *N* is the most significant.

**Figure 5:**
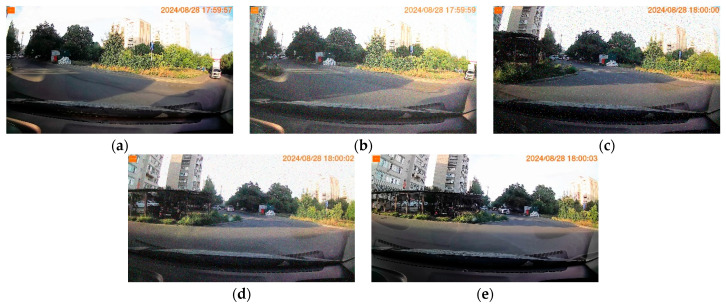
Experimental video frames with various noise types (Example 2): (**a**) Frame 1. (**b**) Frame 50 (Gaussian noise). (**c**) Frame 100 (Impulse noise). (**d**) Frame 150 (Mixed noise). (**e**) Frame 200.

**Figure 6:**
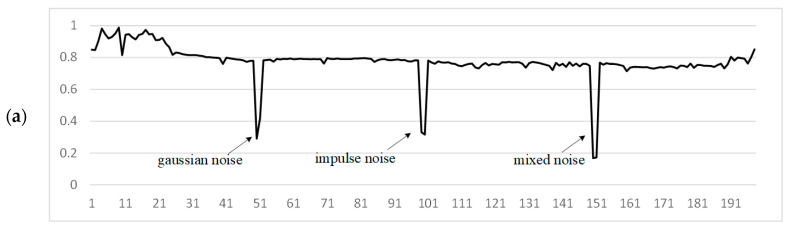

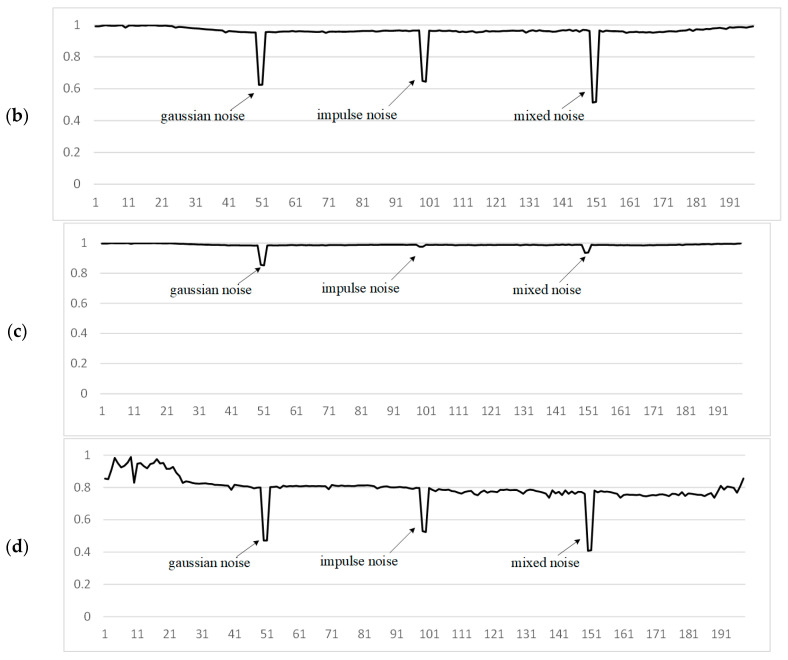
Comparison of SSIM components between video frames: (**a**) *SSIM*; (**b**) *contrast*; (**c**) *lum*; (**d**) *struct*.

**Example 2.**  *Figure 5 shows some frames of a video fragment containing 200 frames, where frame 50 is distorted by Gaussian noise with a density of 0.15; Frame 100 is distorted by impulse noise with a noise density of 0.03; Frame 150 is distorted by impulse noise with a density of 0.01 and is further distorted by Gaussian noise with a density of 0.05. The graphs of these video fragments are shown in Figure 6.*

Considering the brightness factor (Figure 6c), the similarity between video frames is higher than in other aspects, but the presence of a distorted frame is less pronounced. In the structural aspect (Figure 6d), it is more difficult to determine which specific frame is distorted by noise. Based on the values of the SSIM aspects between video frames, the manifestation of additive noise is more pronounced in the contrast aspect (Figure 6b). The original SSIM (Figure 6a) also shows a high result, but a high influence from the differences in other parameters is also observed.

We use the contrast aspect of the SSIM in the proposed approach. To properly use one aspect of SSIM instead of three, the contrast value, calculated by Formula (3), needs to be cubed. The proposed method for evaluating the similarity of video frames will look as follows:

(7) $$S(i-1,i)={\left(\frac{2\sigma_{i-1}\sigma_{i}+C_{2}}{\sigma_{i-1}^{2}+\sigma_{i}^{2}+C_{2}}\right)}^{3}.$$

where $\sigma_{a},\sigma_{b}$ are the standard deviations for *i* − 1 and *i*; $C_{2}$ is a constant similar to Formula (2).

The values of the *S* between consecutive video frames on unmanned vehicles range from [0.9, 1], where 1 indicates complete similarity in the contrast aspect. It is assumed that a value of *S* < 0.9 indicates the presence of noise in the video frame.

**Figure 7:**
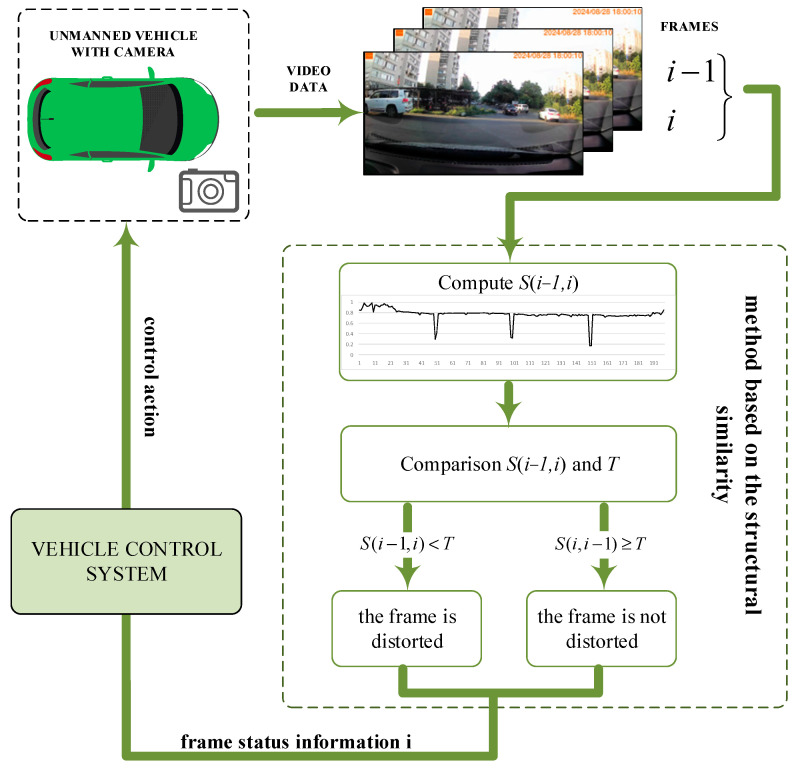
Scheme of the proposed distorted frame detection method for unmanned vehicle video systems.

Figure 7 shows a scheme of the method for detecting distorted frames in video, which uses the proposed method. For a sequence of video frames obtained from the camera of an unmanned vehicle, the proposed method is calculated based on the contrast aspect of the structural similarity index. The value of each *i*-frame of the video is compared with the threshold. Information about the frame state is transmitted to the vehicle control system. Then, the vehicle control system transmits a control action to the unmanned vehicle.

The key requirement for the method is its efficient implementation on onboard UAV processors. The simplicity of the proposed approach (using only the SSIM contrast component) allows achieving low processing time on typical embedded processors. The proposed method, based on the SSIM contrast aspect, allows us to accurately detect the difference between video frames associated with the effect of noise on the image, which makes it promising for solving the problem of detecting distorted video frames. To test its effectiveness and compare it with existing approaches, experiments were conducted on standard datasets. The results of the experiments are given in the next section.

## 4. Materials and Methods

An experimental video was prepared [56] to validate the effectiveness of the proposed method and compare it with other image quality assessment measures for evaluating the similarity of video frames. The video was recorded using the MiDriveD01 dashcam model, containing 1099 frames with a resolution of 1920 × 1080, a frame rate of 30 fps, and in MP4 format. The video frames include OSD information. Every hundredth frame of the experimental video was artificially corrupted. Noise was added after decoding the original video (bitrate 50 Mbps, H.264) to a sequence of uncompressed PNGs, and then controlled noise addition was applied. The simulations and noise processing were performed using MATLAB R2021b. Thus, three copies of the experimental video were created, each differing in the type of noise applied to the corrupted frames. The noise density in the frames increases every hundred frames with a specified step.

Every hundredth frame in Video 1 is corrupted by random impulse noise, with a density ranging from 0.01 to 0.1, increasing in steps of 0.1.

Every hundredth frame in Video 2 is corrupted by Gaussian noise, with a density ranging from 0.05 to 0.5, increasing in steps of 0.05.

Every hundredth frame in Video 3 is corrupted by mixed noise, consisting of a combination of Gaussian noise and random impulse noise.

In the context of digital images, random impulse noise is a kind of noise that can degrade image quality. Sharp, fleeting brightness spikes are one way that this noise can show up at random times. As a result, pixels in the image randomly take on arbitrary values. On the other hand, Gaussian noise is a type of noise characterized by a normal distribution of amplitude values. It often occurs in digital images and can be caused by various factors, such as insufficient lighting, electronic interference, or errors in the image capture process. Gaussian noise is random and unpredictable, making it difficult to remove without losing image details. Since both individual types of noise and combinations of different noise types can occur in real-world conditions, a study was also conducted on mixed noise.

**Figure 8:**
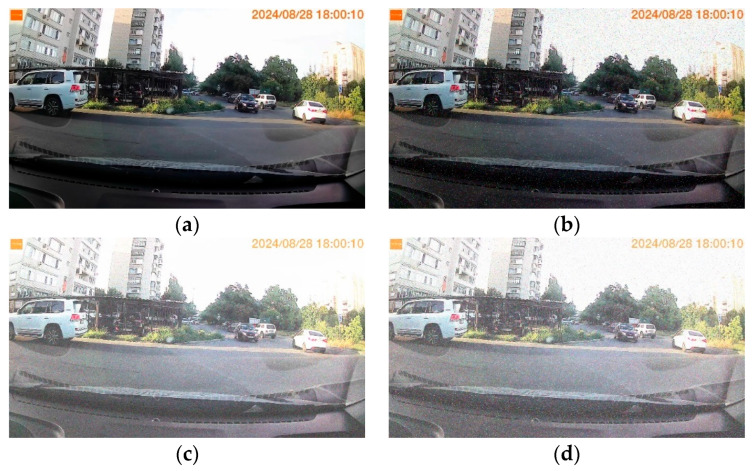
Frame 600 comparison across experimental videos: (**a**) Original undistorted. (**b**) Video 1 (impulse noise). (**c**) Video 2 (Gaussian noise). (**d**) Video 3 (mixed noise).

Figure 8 shows frame 600 from the original undistorted experimental video, Video 1, Video 2, and Video 3. Frame 600 of Video 1 is corrupted by random impulse noise with an intensity of 0.6. Frame 600 of Video 2 is corrupted by Gaussian noise with an intensity of 0.3. Frame 600 of Video 3 is initially corrupted by random impulse noise with an intensity of 0.6, and further corrupted by Gaussian noise with an intensity of 0.3.

**Figure 9:**
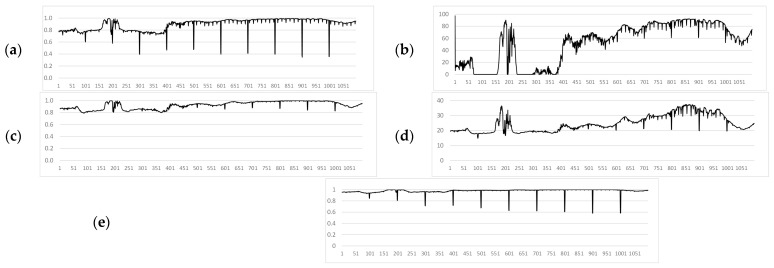
Similarity measures for Video 1 (impulse noise): (**a**) SSIM. (**b**) VMAF. (**c**) CORR. (**d**) PSNR. (**e**) Proposed method (S).

**Figure 10:**
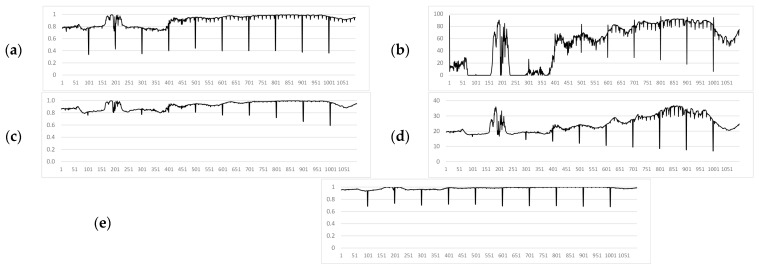
Similarity measures for Video 2 (Gaussian noise): (**a**) SSIM. (**b**) VMAF. (**c**) CORR. (**d**) PSNR. (**e**) Proposed method (S).

**Figure 11:**
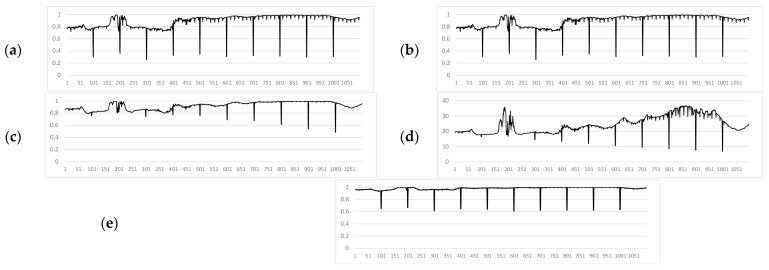
Similarity measures for Video 3 (mixed noise): (**a**) SSIM. (**b**) VMAF. (**c**) CORR. (**d**) PSNR. (**e**) Proposed method (S).

The proposed method was compared with modern approaches for measuring frame similarity to detect inconsistencies. In the experimental videos, the values of the SSIM [42], VMAF [44], CORR [11], PSNR [40] and S (proposed) were calculated. Figure 9, Figure 10 and Figure 11 show the graphs of the values of these measures for Video 1, Video 2, and Video 3, respectively. Measures evaluated on 1099-frame video with artificial noise injected every 100 frames.

A comparative analysis of the similarity measures, as visually summarized in Figure 9, Figure 10 and Figure 11, leads to several key conclusions. The proposed measure *S* (in Figure 9e, Figure 10e and Figure 11e) consistently demonstrates the most pronounced and clear responses to artificially introduced distortions across all noise types (impulse, Gaussian, mixed), with similarity values for corrupted frames dropping sharply below the threshold while remaining stable for uncorrupted segments. In contrast, while SSIM (in Figure 9a, Figure 10a and Figure 11a) shows good detection capability, its response is more susceptible to interference from natural scene dynamics and internal processing artifacts (*F*). VMAF (in Figure 9b, Figure 10b and Figure 11b) exhibits high computational instability, particularly in the initial frames, and fails to provide consistent baseline readings. CORR (in Figure 9c, Figure 10c and Figure 11c), despite its low computational cost, proves ineffective for this task, as its values for distorted frames often do not surpass the variations caused by normal scene changes. Finally, PSNR (in Figure 9d, Figure 10d and Figure 11d) shows poor sensitivity at lower noise levels, making it unreliable for detecting subtle distortions. Thus, the visual evidence from these figures strongly supports the superiority of the proposed contrast-based measure S for the specific task of noise detection in UAV video streams.

An additional measure was introduced to compare image quality methods for determining frame similarity in videos. This measure also allows for an objective assessment of the research results. The measure calculates the arithmetic mean of the absolute differences between the distorted and preceding frames, and is defined as follows.

(8) $$Sens=\frac{1}{10}\left(\sum_{i=1}^{10}\left|S(i-1,i)-S(i-2,i-1)\right|\right).$$

The VMAF takes values in the range [0, 100], PSNR takes values in the range [0,∞]. To compare the Sens values with other measures, it is necessary to normalize these values to the range [0, 1]. PSNR can be normalized to the range [0, 1] by dividing the value by 40, since 40 dB corresponds to high image quality where distortion is visually imperceptible [45]. This linear normalization approach is widely adopted in image quality assessment literature to provide an intuitive scaling where values near 1 represent almost distortion-free content, while lower values indicate progressively more severe degradation. The threshold of 40 dB represents the point where distortions typically become imperceptible to human observers under normal viewing conditions, making it appropriate for establishing an upper bound for quality assessment in our video analysis context. The Sens allows determining the sensitivity of detecting a distorted frame using the corresponding method (SSIM, VMAF, CORR, PSNR, *S*). The *Sens* was calculated for Video 1, Video 2, and Video 3.

The SSIM demonstrated high effectiveness in detecting distorted frames in the video. However, the graph reveals a significant influence of *F*, which is attributed to the periodic activation of internal algorithms of the video recorder. The computation of the VMAF demands greater computational resources. Additionally, the VMAF exhibits instability during the first 400 frames of the video due to its use of machine learning methods. This instability may be associated with the training datasets used in the VMAF model. A key advantage of the CORR is its low computational demand and energy efficiency. However, in all experimental videos, this method showed low effectiveness, with the CORR values under strong *N* influences not exceeding the effect of *D*. Consequently, it is not possible to reliably determine whether a frame is distorted. At low noise levels, PSNR fails to detect distorted frames by comparing successive frames, as can be seen from Table 2. Sensitivity values calculated using Equation (8), representing the mean absolute difference between distorted and preceding frames. For this reason, PSNR showed detection results similar to VMAF.

The proposed method accurately detects all 10 artificially corrupted frames despite the different types of distortions. In Video 1, as the intensity of the random impulse noise increases, the detection of distorted frames becomes more effective. However, in Video 2 and Video 3, the presence of all distorted frames is evident, but as the noise intensity increases, the detection efficiency does not improve. It is also clear that, for the proposed measure S, the similarity between the uncorrupted frames does not exceed the threshold *T* throughout the entire video.

Also, the proposed method accurately detects all 10 artificially distorted frames despite the diverse nature of distortions. In Video 1, as the intensity of random impulse noise increases, the detection of distorted frames becomes more efficient. In contrast, in Videos 2 and 3, although the presence of all distorted frames is evident, the detection efficiency does not improve with the rise in distortion intensity. Additionally, for the proposed measure *S*, it is observed that the similarity values between undistorted frames do not exceed the threshold *T* throughout the entire video sequence.

The obtained Sens values confirm the conclusions drawn from the plots in Figure 7, Figure 8 and Figure 9 and demonstrate the superiority of the proposed method across all experimental videos. The highest Sens values achieved by the proposed method indicate a more pronounced presence of distortions in the video frames. Therefore, the proposed method effectively detects distorted frames in videos captured by unmanned vehicles.

**Figure 12:**
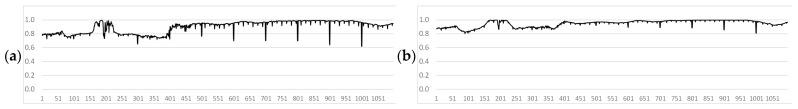
Performance comparison for overexposure distortion: (**a**) SSIM. (**b**) Proposed method (S).

One of the limitations of the proposed method, which is based solely on the contrast component of the SSIM index, is its inability to detect frame distortions caused by sensor overexposure, such as glare from direct sunlight. To model this scenario, a simulation was conducted in which synthetic “glare” distortions were introduced into a video sequence by artificially increasing pixel brightness values. Specifically, every 100th frame was modified by increasing pixel intensity: by +10 for frame 100, +20 for frame 200, and so on, up to +100 for frame 1000. Since the developed similarity measure excludes the luminance and structural components of SSIM, the method fails to detect frames affected by overexposure. As a result, despite noticeable brightness changes, such frames are not identified as distorted because the metric relies solely on contrast. This experiment highlights a key limitation: the method is not effective in scenarios where brightness is the dominant form of distortion, as it relies exclusively on contrast variation. The results of this simulation are presented in Figure 12.

Another important scenario to consider is when distorted frames appear consecutively rather than being isolated. Table 3 presents the values of the proposed contrast-based similarity measure in cases where adjacent frames are corrupted by Gaussian and random impulse noise. Frames 100, 101 and 102 have the same density of random impulse and Gaussian noise. The results indicate that the method is capable of detecting such frames, as the similarity values remain below the threshold. However, this observation does not imply that the proposed approach is universally effective for all types of noise when distortions occur in sequence. Nevertheless, it can be reliably stated that the proposed method will consistently detect the first distorted frame in a sequence, as its contrast with the preceding undistorted frame is always significant. It is also worth noting that the frame following the distorted one will have low similarity values, since it is very different from the distorted frame. In this case, frame 103 is not distorted, but has low *S* and *SSIM* values.

**Figure 13:**
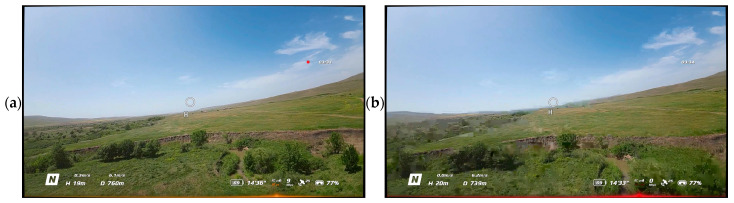
Video frames from the DJI Avata UAV (999 frames): (**a**) An example of an undistorted video frame. (**b**) An example of a distorted video frame.

**Figure 14:**
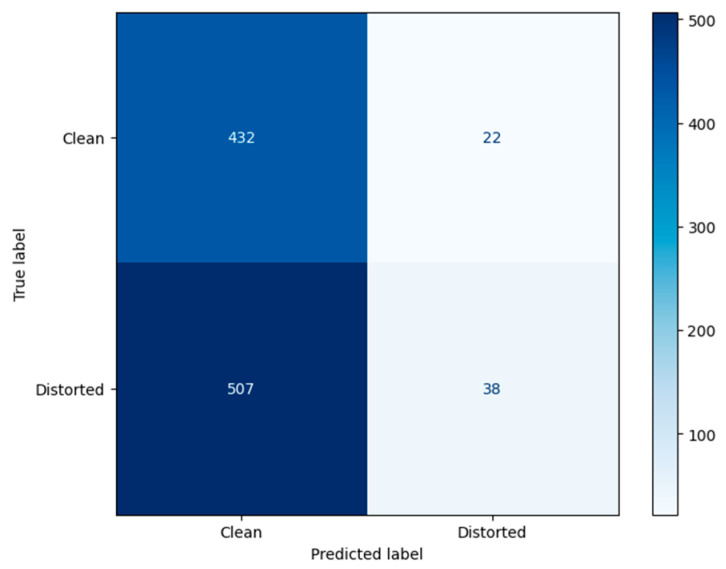
Confusion matrix for the binary classification of video frames as “distorted” or “clean” using the proposed method with a threshold of *T* = 0.9 for video from the DJI Avata UAV with non-artificial distortions.

**Figure 15:**
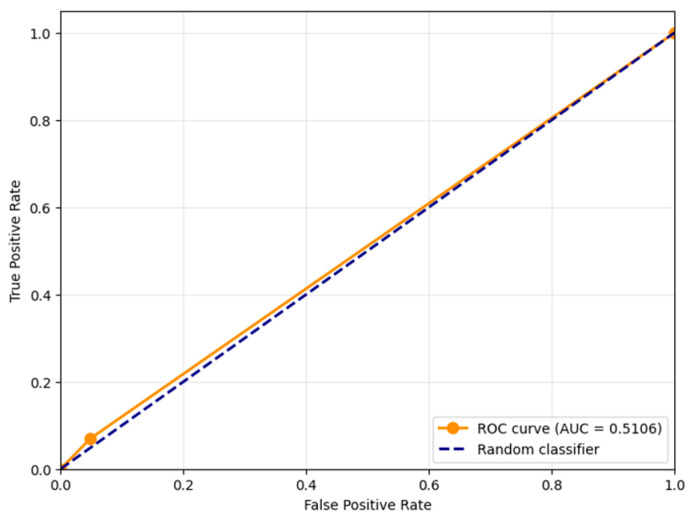
Receiver Operating Characteristic (ROC) curve for the proposed distortion detection method, with the Area Under the Curve (AUC) for video from the DJI Avata UAV with non-artificial distortions.

To test the robustness of the proposed method under conditions as close as possible to those described in UAV operation, an additional experiment was conducted. The test data consisted of video recorded from a DJI Avata UAV, which exhibited characteristic artifacts caused by signal loss: compression artifacts (blocking effect) and time delays (frame dependency and duplication). These interpretations were not artificially simulated but occurred during a real flight, making them valuable for assessing the practical applicability of the method. Examples of video frames are shown in Figure 13. Figure 14 presents the confusion matrix for the binary classification of video frames as “distorted” or “clean” using the proposed method with a threshold of *T* = 0.9. The values of the metrics Accuracy, Precision, Recall, F1-Score are presented in Table 4. Figure 15 shows the ROC curve for the proposed distortion detection method, with the AUC.

The application of the proposed method to real-world UAV footage exhibiting compression artifacts and lag revealed a fundamental limitation of the contrast-based approach. The classification results on this dataset (Accuracy = 0.47, Precision = 0.63, Recall = 0.07) demonstrate that the method is not effective for this class of distortions (Figure 14). The critically low Recall value of 0.07 indicates that the model fails to detect the vast majority of actual distorted frames, while the moderate Precision of 0.63 shows that even the few detections made are unreliable. This conclusion is further reinforced by the ROC-AUC value of 0.5106 (Figure 15), which is virtually equivalent to random guessing (AUC = 0.5). This statistically confirms that the proposed metric S possesses no meaningful discriminative power for this specific distortion type and cannot reliably separate corrupted and clean frames under compression artifacts. This outcome is analytically consistent: compression artifacts primarily degrade structural information and cause global frame shifts during lag, to which the contrast component of SSIM is inherently less sensitive compared to additive noise. Therefore, these results serve not to discredit the method but to precisely define its operational domain. They conclusively show that the proposed technique is a specialized tool for detecting noise corruption in high-integrity video streams, and is not suited for diagnosing bandwidth-related artifacts like compression or lag. This finding is of significant practical importance for system architects, as it clarifies that different distortion types in UAV video pipelines require distinct, specialized detection mechanisms.

## 5. Discussion

SSIM and VMAF were originally developed for other tasks, but their adaptation to noise detection yields good results. Although these metrics were actually created to assess visual quality from a human perspective, the underlying criteria they establish, such as structural similarity, visibility convention, and spatial cues, are essentially objective and original representations of the data. SSIM analyzes local features of the pixel brightness distribution and their relationships—these are mathematical properties of the image, not abstract phenomena. Similarly, VMAF observes not only human perceptual patterns, but also low-level features such as contrast and edge preservation. These parameters retain their innovativeness even when the «consumer» of the frames is the method, not the observer.

The approach presented in [1], which employs correlation for detecting edits in videos by comparing frames, does not require high computational resources but has not demonstrated satisfactory results. The detection of distorted video frames using the method described in [11] is feasible only under conditions of very low influence of *D* in the video, making it practically ineffective. The VMAF [49] is capable of detecting distorted frames. However, because it is based on machine learning methods, certain segments of the video produce low measure values that do not reflect reality.

Among the methods considered in Section 4, the proposed method and SSIM [47] demonstrated the best performance, as evidenced by the numerical results in Table 1. The superiority of the proposed method is attributed to its focus on the contrast aspect, where the influence of *N* is most pronounced, as illustrated in Figure 2. Videos captured by unmanned vehicles typically do not include edits or superimposed graphics. Contrast in such videos generally falls within a narrow range of values, unlike brightness and structural elements. It is necessary to calculate the values of the sentence (Formula (7)) for the received video from an unmanned vehicle. A conclusion is made for the distortion of this video frame when comparing the obtained measure values with the threshold value.

Although three types of noise are considered in the work, including random impulse, Gaussian and mixed, there are other types of distortions that can significantly affect the video received from unmanned vehicles. For example, compression artifacts, blurry images, as well as glare from light sources can decrease the quality of the video. These distortions are not considered in this article. Presumably, any type of distortion affects the structural similarity of images, including in the aspect of contrast. In the future, it is advisable to investigate the stability of the proposed method to such distortions and the ability to expand the method for a wider noise spectrum.

It should also be noted that the analysis showed that the values of the proposed method can be affected by periodic activation of the built-in DVR algorithms (*F*). This indicates the possible sensitivity of the method to the specific equipment and firmware used in the system. At the same time, Figure 2 shows that such fluctuations are insignificant and cannot practically be identified as noise. It is important to take into account that the results may vary depending on the DVR model. The level of influence of distortions (*F*) is much less than the influence of even 1% of noise. This means that in practice, noise *F* can be neglected.

The fixed threshold of S < 0.9 was empirically determined through a comprehensive analysis of ROC curves and precision-recall trade-offs across our experimental datasets. This value demonstrated an optimal balance between detection sensitivity and false positive rates, achieving maximal F1-scores while maintaining practical applicability for real-time systems. However, the generalizability of this specific threshold value across different camera models and environmental conditions requires careful consideration. Variations in sensor characteristics, lens properties, automatic exposure adjustments, and native image processing pipelines between camera systems may systematically affect the absolute values of the contrast-based similarity measure. Similarly, environmental factors such as lighting conditions, weather, and scene dynamics could influence the baseline similarity between consecutive frames. Therefore, while the methodological approach remains universally applicable, the optimal threshold value may benefit from camera-specific calibration or adaptive adjustment based on operational conditions. Future work should explore adaptive thresholding strategies that dynamically adjust to changing environmental contexts and camera-specific characteristics to ensure robust performance across diverse UAV platforms and mission profiles.

While the present study does not include a detailed runtime analysis, the computational efficiency of the proposed method follows directly from its algorithmic simplicity. Using only the contrast component of SSIM eliminates the need to compute the luminance and structural components, reducing the number of required mathematical operations by approximately three times compared to the full SSIM version. This approach is particularly important for resource-constrained systems, where even a minor reduction in computational load can be critical for enabling real-time operation. A promising direction for future research is the precise quantitative evaluation of the method’s performance on various hardware platforms used in unmanned systems.

The proposed method may seem simple, but its value lies in adapting the classical SSIM to the specific task of noise detection in videos from unmanned vehicles. This allows achieving high accuracy at low computational cost, which is critical for embedded systems. The approaches and methods discussed in this paper are applicable to cameras used in unmanned ground, water, and aerial vehicles. Different types of unmanned vehicles have varying constraints on energy consumption and computational resources. The approaches from [12,47] and the proposed method are suitable for unmanned vehicles with limited device dimensions. In contrast, employing the VMAF [48] in unmanned aerial vehicles would result in rapid battery depletion. Future research could focus on implementing adaptive thresholding techniques could refine distortion detection, making it more effective for real-time applications in unmanned systems.

## 6. Conclusions

This paper proposes an efficient method for detecting distortions in videos captured by unmanned vehicles, based on analyzing the contrast component of the Structural Similarity Index (SSIM) between consecutive frames. Experimental results demonstrate that the proposed method effectively identifies distortions caused by various types of noise, such as random impulse, Gaussian, and mixed noise. Notably, the method requires minimal computational resources and has a negligible impact on the energy efficiency of unmanned vehicle recording systems.

In this work, the classical SSIM approach is specifically adapted for detecting distorted frames in UAV video by isolating its most responsive component—contrast. The standard SSIM components were reweighted to reflect the higher sensitivity of the contrast element to typical distortions observed in unmanned vehicle footage.

Furthermore, an evaluation based on the mean absolute difference in similarities between frames objectively demonstrates the detection accuracy of the proposed method. The findings of this study are of substantial practical importance for systems that rely on video recordings from unmanned vehicles, where high video quality is critical for the accuracy of subsequent data processing and analysis.

## Figures and Tables

**Table 1:** Performance metrics for the proposed distortion detection method at the selected threshold of *T* = 0.9.

	Video
Accuracy	0.9909
Precision	0.5000
Recall	1.0000
F1-Score	0.6667

**Table 2:** Accuracy of similarity measures for Video 1, Video 2 and Video 3 using Formula (8).

	Video 1	Video 2	Video 3
Proposed measure S	0.6256	0.6141	0.7029
SSIM [47]	0.4549	0.5157	0.5933
VMAF [49]	0.1138	0.3409	0.3974
CORR [11]	0.0738	0.1793	0.2440
PSNR [45]	0.1251	0.1939	0.3423

**Table 3:** Detection results for consecutive frames distorted by impulse and Gaussian noise.

Frame Number		99	100	101	102	103	104
Random impulse noise	Proposed measure S	0.8342	0.6380	0.2633	0.2345	0.1458	0.8378
	SSIM [47]	0.7842	0.6428	0.1880	0.1346	0.2076	0.7873
Gaussian noise	Proposed measure S	0.8342	0.3146	0.5516	0.2298	0.1458	0.8378
	SSIM [47]	0.7842	0.3438	0.1460	0.1333	0.2076	0.7873

**Table 4:** Performance metrics for the proposed distortion detection method at the selected threshold of *T* = 0.9 for video from the DJI Avata UAV with non-artificial distortions.

	Video from the DJI Avata UAV
Accuracy	0.4705
Precision	0.6333
Recall	0.0697
F1-Score	0.1256

## Data Availability

The data presented in this study are openly available in https://bitbucket.org/anzor_orazaev/d_video/src/main (accessed on 23 October 2025), reference [56].

## References

[1] Shakhatreh H., Sawalmeh A.H., Al-Fuqaha A., Dou Z., Almaita E., Khalil I., Othman N.S., Khreishah A., Guizani M. (2019). Unmanned aerial vehicles (UAVs): A survey on civil applications and key research challenges. IEEE Access.

[2] Kujawski A., Dudek T. (2021). Analysis and visualization of data obtained from camera mounted on unmanned aerial vehicle used in areas of urban transport. Sustain. Cities Soc..

[3] Dai Y., Wu Z., Wang F., Zhang Y. (2022). A survey of detection-based video multi-object tracking. Displays.

[4] Estrada M.A.R., Ndoma A. (2019). The uses of unmanned aerial vehicles–UAVs–(or drones) in social logistic: Natural disasters response and humanitarian relief aid. Procedia Comput. Sci..

[5] Bhagat B.B., Sharma R.R., Tilante D. (2023). Moving camera-based automated system for drone identification using focus measures. Signal Image Video Process..

[6] Li G., Zhang X., Wang Z., Li X., Zhang Y. (2024). Lightweight wildfire smoke monitoring algorithm based on unmanned aerial vehicle vision. Signal Image Video Process..

[7] Fu F., Kang Y., Zhang Z., Yu F.R., Wu Q. (2023). Live traffic video multicasting services in UAV-assisted intelligent transport systems: A multiactor attention critic approach. IEEE Internet Things J..

[8] Liu H., Li X., Zhou G., Liu Y., Li M., Zhang Z., Zhang Y., Wu J., Yu N. (2023). Coherent adversarial deepfake video generation. Signal Process..

[9] Qu Z., Li J., Gao L. (2022). A method of image stitching with partition matching and direct detection for rotated image. Displays.

[10] Wu J., Cui Z., Sheng V.S., Zhao P., Su D., Gong S. (2013). A Comparative Study of SIFT and its Variants. Meas. Sci. Rev..

[11] Fayyaz M.A., Sharif M., Raza M., Saba T., Rehman A., Iqbal T. (2020). An improved surveillance video forgery detection technique using sensor pattern noise and correlation of noise residues. Multimed. Tools Appl..

[12] Oyallon E., Rabin J. (2015). An analysis of the SURF method. Image Process. Online.

[13] Bansal M., Kumar M., Kumar M. (2021). 2D object recognition: A comparative analysis of SIFT, SURF and ORB feature descriptors. Multimed. Tools Appl..

[14] Niu B., Gao Z., Guo B. (2021). Facial expression recognition with LBP and ORB features. Comput. Intell. Neurosci..

[15] Shi L., Wang X., Shen Y. (2020). Research on 3D face recognition method based on LBP and SVM. Optik.

[16] Campos C., Elvira R., Rodríguez J.J.G., Montiel J.M.M., Tardós J.D. (2021). ORB-SLAM3: An accurate open-source library for visual, visual–inertial, and multimap slam. IEEE Trans. Robot..

[17] Viswanathan D.G. Features from accelerated segment test (FAST). Proceedings of the 10th Workshop on Image Analysis for Multimedia Interactive Services.

[18] Calonder M., Lepetit V., Strecha C., Fua P. (2010). BRIEF: Binary robust independent elementary features. Proceedings of the Computer Vision—ECCV 2010: 11th European Conference on Computer Vision.

[19] Setiawan A., Yunmar R.A., Tantriawan H. (2020). Comparison of speeded-up robust feature (SURF) and oriented FAST and rotated BRIEF (ORB) methods in identifying museum objects using low light intensity images. IOP Conf. Ser. Earth Environ. Sci..

[20] Kazerouni I.A., Fitzgerald L., Dooly G., Toal D. (2022). A survey of state-of-the-art on visual SLAM. Expert. Syst. Appl..

[21] Siddique N., Sidike P., Elkin C., Devabhaktuni V. (2021). U-net and its variants for medical image segmentation: A review of theory and applications. IEEE Access.

[22] Haddad J., Lézoray O., Hamel P. (2020). 3D-CNN for facial emotion recognition in videos. Proceedings of the Advances in Visual Computing: 15th International Symposium, ISVC 2020.

[23] Tariq S., Lee S., Woo S.S. (2020). A convolutional LSTM-based residual network for deepfake video detection. arXiv.

[24] Tipper S., Atlam H.F., Lallie H.S. (2024). An investigation into the utilisation of CNN with LSTM for video deepfake detection. Appl. Sci..

[25] Saikia P., Dholaria D., Yadav P., Patel V., Roy M. A hybrid CNN-LSTM model for video deepfake detection by leveraging optical flow features. Proceedings of the 2022 International Joint Conference on Neural Networks (IJCNN).

[26] Duan H., Hu Q., Wang J., Yang L., Xu Z., Liu L., Min X., Cai C., Ye T., Zhang X. (2024). FineVQ: Fine-Grained User Generated Content Video Quality Assessment. arXiv.

[27] Kumar R., Corvisieri G., Fici T.F., Hussain S.I., Tegolo D., Valenti C. (2025). Transfer Learning for Facial Expression Recognition. Information.

[28] Das A.K., Leung C.K.Y. (2024). A Novel Technique for High-Efficiency Characterization of Complex Cracks with Visual Artifacts. Appl. Sci..

[29] Das A.K., Leung C.K.Y. (2025). A novel deep learning-based technique for efficient characterization of engineered cementitious composites cracks for durability assessment. Struct. Concr..

[30] Zhang R., Xu L., Yu Z., Shi Y., Mu C., Xu M. (2021). Deep-IRTarget: An automatic target detector in infrared imagery using dual-domain feature extraction and allocation. IEEE Trans. Multimed..

[31] Zhang J., Zhang R., Xu L., Lu X., Yu Y., Xu M., Zhao H. (2024). Fastersal: Robust and real-time single-stream architecture for RGB-D salient object detection. IEEE Trans. Multimed..

[32] Liu H., Klomp N., Heynderickx I. (2010). A no-reference metric for perceived ringing artifacts in images. IEEE Trans. Circuits Syst. Video Technol..

[33] Gardikis G., Boula L., Xilouris G., Kourtis A., Pallis E., Sidibé M., Négru D. (2012). Cross-layer monitoring in IPTV networks. IEEE Commun. Mag..

[34] Zhang L., Zhang L., Bovik A.C. (2015). A feature-enriched completely blind image quality evaluator. IEEE Trans. Image Process..

[35] Wu L., Yan Y., Zhang L., Wu J., Xie Y., Zhang Y., Fan H. (2021). VP-NIQE: An opinion-unaware visual perception natural image quality evaluator. Neurocomputing.

[36] Zvezdakova A., Erofeev M., Vatolin D. (2019). Barriers towards no-reference metrics application to compressed video quality analysis: On the example of no-reference metric NIQE. arXiv.

[37] Mittal A., Moorthy A.K., Bovik A.C. (2012). No-reference image quality assessment in the spatial domain. IEEE Trans. Image Process..

[38] Chow L.S., Rajagopal H. (2017). Modified-BRISQUE as no reference image quality assessment for structural MR images. Magn. Reson. Imaging.

[39] Gavrovska A., Pavlovic V., Milivojevic M., Reljin I., Reljin B. No-reference local image quality evaluation. Proceedings of the 2022 30th Telecommunications Forum (TELFOR).

[40] Pandey A., Kumar P., Malhotra A., Rastogi A., Yadav D.K., Mittal B.R. (2020). Evaluation of Perception Based Image Quality Evaluator (PIQE) no-reference image quality score for 99mTc-MDP bone scan images. J. Nucl. Med. Technol..

[41] Mo L., Wang K., Li Y., Wang S., Wang Z. (2024). A no-reference video quality assessment method with bidirectional hierarchical semantic representation. Signal Process..

[42] Xiang J., Jiang Z., Yu L., Wang F., Gu K., Jiang G. (2023). Blind light field image quality assessment with tensor color domain and 3D shearlet transform. Signal Process..

[43] Egiazarian K., Astola J., Ponomarenko N., Lukin V., Battisti F., Carli M. New full-reference quality metrics based on HVS. Proceedings of the Second International Workshop on Video Processing and Quality Metrics.

[44] Hore A., Ziou D. Image quality metrics: PSNR vs. SSIM. Proceedings of the 2010 20th International Conference on Pattern Recognition.

[45] Gonzalez R.C., Woods R.E. (2018). Digital Image Processing.

[46] Pedersen M., Hardeberg J.Y. (2012). Full-reference image quality metrics: Classification and evaluation. Found. Trends Comput. Graph. Vis..

[47] Wang Z., Bovik A.C., Sheikh H.R., Simoncelli E.P. (2004). Image quality assessment: From error visibility to structural similarity. IEEE Trans. Image Process..

[48] Lyakhov P., Orazaev A. (2025). Analysis of video data of an unmanned aerial vehicle based on the structural similarity index. Computers. Optics..

[49] Li Z., Aaron A., Katsavounidis I., Moorthy A., Bovik A. (2018). VMAF: The journey continues. Netflix Technol. Blog.

[50] Rassool R. VMAF reproducibility: Validating a perceptual practical video quality metric. Proceedings of the 2017 IEEE International Symposium on Broadband Multimedia Systems and Broadcasting (BMSB).

[51] Deng S., Han J., Xu Y. VMAF-based rate-distortion optimization for video coding. Proceedings of the 2020 IEEE 22nd International Workshop on Multimedia Signal Processing (MMSP).

[52] Min X., Duan H., Sun W., Zhu Y., Zhai G. (2024). Perceptual Video Quality Assessment: A Survey. arXiv.

[53] Shen J., Jiang X., Zhong J., Yao S. Scene change detection based on sequence statistics using structural similarity. Proceedings of the 2022 4th International Academic Exchange Conference on Science and Technology Innovation (IAECST).

[54] Yedla S.K., Manikandan V.M., Panchami V. Real-time Scene Change Detection with Object Detection for Automated Stock Verification. Proceedings of the 2020 5th International Conference on Devices, Circuits and Systems (ICDCS).

[55] Gupta L., Jain R., Vaszkun G. (2016). Survey of important issues in UAV communication networks. IEEE Commun. Surv. Tutor..

[56] Video for Modeling. https://bitbucket.org/anzor_orazaev/d_video/src/main.

